# Family functionality as a mediator in the relationship between humanization and academic burnout in adolescents

**DOI:** 10.3389/fpsyg.2024.1520912

**Published:** 2025-01-15

**Authors:** Pablo Molina Moreno, Silvia Fernández Gea, María del Carmen Pérez-Fuentes, María del Mar Molero Jurado, José Jesús Gázquez Linares

**Affiliations:** ^1^Department of Psychology, Universidad de Almería, Almería, Spain; ^2^Department of Psychology, Universidad Autónoma de Chile, Santiago, Chile

**Keywords:** personal competencies, family functionality, academic burnout, humanization, adolescence, education, high-school students

## Abstract

**Introduction:**

During adolescence, personal competencies serve as protective factors against social exclusion and are crucial for promoting psychological well-being and creating opportunities for growth. Family and educational systems play a pivotal role in supporting these competencies. This study aims to analyze the relationships between humanization competencies, academic burnout, and family functionality, to examine sex differences in these variables, and to explore the mediating effect of family functionality.

**Methods:**

The sample comprised 1,092 secondary school students (584 females and 508 males) from Almería, Spain, aged 12 to 17 (*M* = 14.5 years).

**Results:**

The findings show that family functionality is positively associated with humanization competencies and academic efficacy, and negatively associated with emotional exhaustion and cynicism. Notable sex differences emerged, with males scoring higher in optimism, self-efficacy, and affect, while females scored higher in sociability and cynicism. Mediation analysis revealed that family functionality partially mediates the relationship between humanization competencies and academic burnout, specifically impacting cynicism, academic efficacy, and emotional exhaustion.

**Discussion:**

These results highlight the critical role of a supportive family environment in enhancing adolescents’ psychological resilience and academic outcomes. The study suggests that interventions targeting family functionality could be effective in reducing academic burnout and promoting overall well-being among adolescents.

## 1 Introduction

Adolescence is the developmental period between childhood and adulthood, marked by the onset of puberty and ending when an individual achieves independence within society (Dumontheil, 2016; Sibilia et al., 2024). The World Health Organization (World Health Organization, 2021) defines this stage as occurring between the ages of 10 and 19, emphasizing its significance due to the relevant emotional, social and physical changes experienced by adolescents, which place them in a vulnerable position to factors that may impair their development towards adulthood. Studies such as that of Tervo-Clemmens et al. (2024) highlight this stage as one characterized by heightened exposure to risk behaviors. Having a well-developed set of personal competencies during adolescence is crucial for effectively managing these challenges and promoting psychological well-being (Schoeps et al., 2021). These personal competencies protect against social exclusion and rejection (Griffin, 2017) during a stage when social relationships become more complex, driven by developing executive functions and heightened social sensitivity—key influences on adolescent development (Blakemore and Mills, 2014).

Therefore, to ensure the comprehensive well-being of adolescents, it is essential to prioritize the improvement of their mental health and psychological aspects (Matić and Musil, 2023). Numerous studies highlight the importance of cultivating personal competencies to enhance the well-being of young individuals (Galván, 2015; Molina-Montes et al., 2023). Some researchers conceptualize these personal skills under the framework of “humanization,” which includes competencies such as Disposition to Optimism, Emotional Understanding, Sociability, Affect, and Self-Efficacy (Pérez-Fuentes et al., 2019a,b). The development of these competencies from an early age should be supported by family and educational systems to strengthen youth, thereby promoting greater overall well-being and expanding future opportunities (Márquez and Gaeta, 2017).

Insufficient development of these competencies can lead to lower academic performance and increased stress in students (Llanos, 2016; Pérez and Chávez, 2024). Burnout is a condition that arises after prolonged exposure to adverse events, such as chronic stress, and manifests through physical, psychological, and emotional symptoms (Abarkar et al., 2023). When the origin of stress is in the school setting, this disorder is referred to as academic burnout and is identified as a persistent and negative psychological state experienced by students in relation to their learning (Gao, 2023). Adolescents who experience academic burnout usually present physical exhaustion and emotional exhaustion; feelings of cynicism towards their studies, understood as indifference or lack of interest; and low levels of academic efficacy, which refers to the aptitude for the performance of their academic competencies (Usán et al., 2020). Although this type of disorder can affect each adolescent differently, it has been evidenced in works such as Lee and Lee (2018) and Barbosa-Camacho et al. (2022), that academic burnout can affect the individual’s relationships with their teachers, family and friends; as well as their attitudes towards study, negatively affecting academic performance, adaptation to school life and satisfaction with life. Ultimately, academic burnout and social anxiety can lead to problem behaviors such as absenteeism and dropping out of school (Sasagawa and Essau, 2022; Wang et al., 2019), given the lack of participation in class activities, frequent absences, and feelings of meaninglessness and incompetence in learning content (Rezaei et al., 2021).

Stressful academic events increase the probability that high school students present symptoms of depression (Salmela-Aro, 2022) and anxiety (Gázquez et al., 2023; Martínez-Líbano et al., 2022). In addition, adolescents’ mental health symptoms, such as depression and anxiety, are closely linked to higher levels of academic burnout, as they tend to rely on avoidance strategies due to a lack of positive emotional regulation techniques (Vinter et al., 2021). Additionally, a strong sense of academic self-efficacy positively influences academic performance and school adaptation, which, in turn, enhances overall life satisfaction (Castelli and Marcionetti, 2024; Kim and Park, 2020).

Although the development of academic burnout syndrome is influenced by individual factors, the influence of the adolescent’s family factors has also been argued (Liu et al., 2024; Zhang et al., 2023), an aspect highlighted in studies such as that of Wu et al. (2022) where the existence of a significant correlation between academic burnout and family functioning has been highlighted. Family functionality refers to the growth and maturation, both physically and psychologically, of all the members that make up a family, which is a relevant factor for adolescents as it affects their overall behavior (Wu et al., 2016). Affective bonds within a functional family can help it adapt to challenges, fostering higher adolescent satisfaction, whereas family dysfunction reduces satisfaction and increases vulnerability in physical, psychological, and social development (Mateo-Crisóstomo et al., 2018). It has been seen that good family functionality acts as a protective factor against risk behaviors, such as difficulties in communicating with friends, self-esteem problems and decision-making (Esteves et al., 2020), given that the affective relationships that are generated provide a social support network and an increase in adaptive capacity through emotional regulation skills (Barragán et al., 2021). This fosters a greater ability to overcome adverse situations, mitigating mental health issues such as anxiety and depression (Elizarov et al., 2023), reducing exposure to tobacco and alcohol consumption (Molero et al., 2019; Musitu et al., 2007), and lowering the likelihood of developing psychopathic traits (Cano-Lozano et al., 2023; Jiménez-Granado et al., 2023).

Similarly, personal competencies such as self-efficacy (Zakiei et al., 2020), affect (Alvarez-Subiela et al., 2022), sociability (Esteves et al., 2020), emotional skills (Szcześniak and Tułecka, 2020) and optimism (Barragán et al., 2021) have been directly related to family functionality.

Additionally, it is necessary to consider adolescents’ personal characteristics, such as age and sex, as these factors play a key role in empirical studies examining humanization competencies (Bermejo et al., 2013; Villacieros et al., 2019), academic burnout (Read et al., 2022; Vinter et al., 2021), and family functionality (Barragán et al., 2021; Spitz and Steinhausen, 2023).

Family functionality has been identified as a mediator in various adolescent contexts, such as the relationship between prosocial behavior and school climate (González and Molero, 2023). However, there is a limited body of research that has specifically explored the mediating effect between personal competencies and academic burnout in depth, highlighting the need for further investigation in this area. Consequently, the main objectives of the present study are: (1) to analyze the relationships between humanization competencies, academic burnout, and family functioning in adolescents; (2) to explore potential sex differences in these variables; and (3) to examine the mediating role of family functioning in the relationship between humanization competencies and academic burnout.

**Figure 1:**
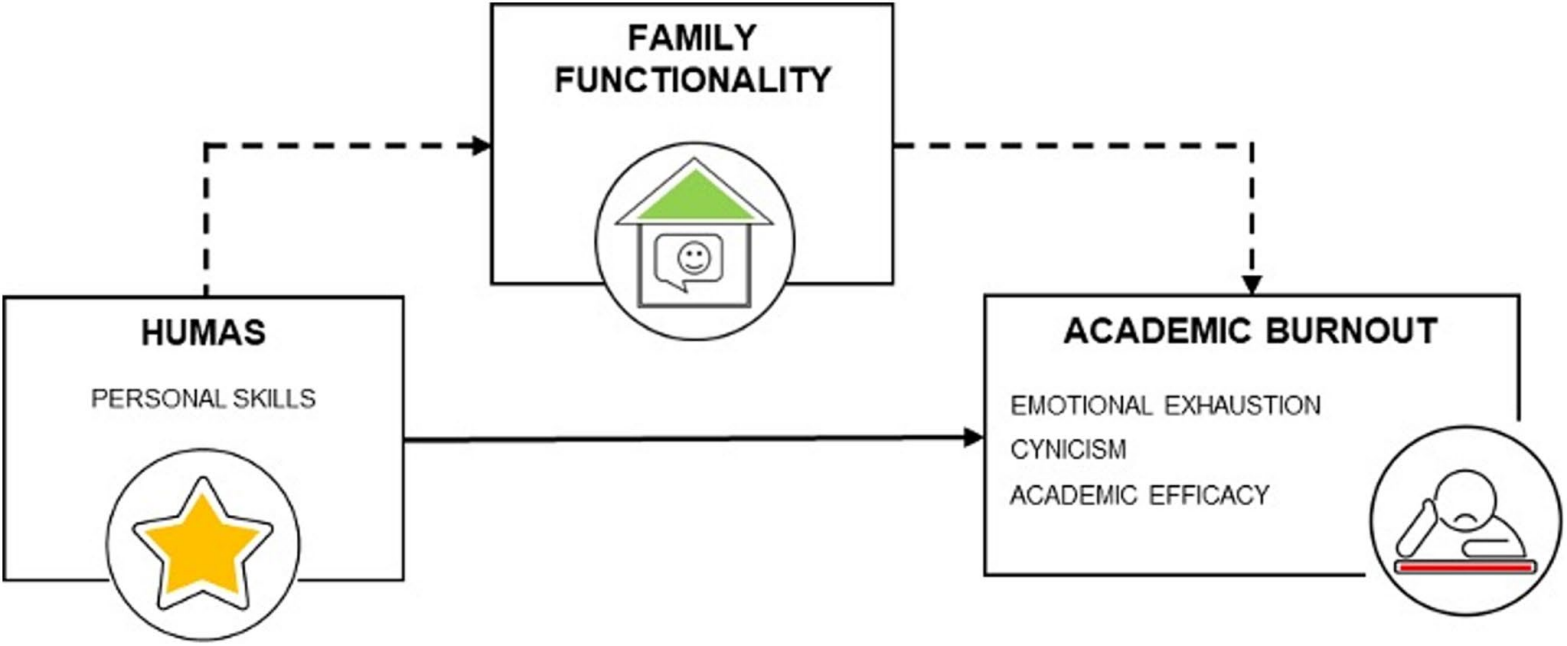
Hypothesized mediation model. Source: Own elaboration.

The following are the research hypotheses established for this study. The first hypothesis (H1) seeks to establish positive associations between the three primary variables: humanization competencies, academic burnout and family functionality. The second hypothesis (H2) focuses on investigating whether there are significant sex differences in humanization, academic burnout and family functioning. And the third hypothesis (H3) explores whether family functionality acts as a mediator in the relationship between personal competencies (HUMAS) and the dimensions of academic burnout in adolescents (Figure 1).

## 2 Materials and method

### 2.1 Participants

To select the participants for the sample and ensure the study’s relevance and consistency, specific inclusion and exclusion criteria were established to the inclusion criteria were: (a) enrollment in a secondary education institution, (b) age between 12 and 16 years, and (c) possession of signed informed consent from parents or legal guardians. Conversely, the exclusion criteria included: (a) any pre-existing medical or psychological condition that could interfere with the accurate completion of the questionnaires, and (b) failure to complete the questionnaire in its entirety, as incomplete data were excluded from the analysis. These criteria ensured a homogeneous sample of adolescents within the school context, aligned with the study’s objectives.

**Figure 2:**
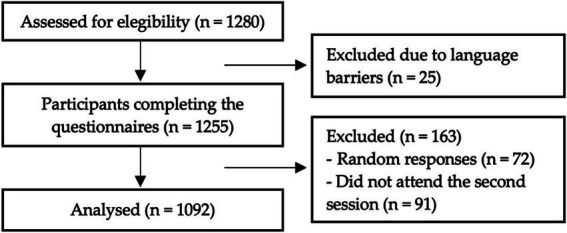
Flowchart of the final sample selection process. Source: Own elaboration.

This study involved a final sample of 1,092 secondary school students from the province of Almería, Spain, drawn from six high schools and spanning the 2nd, 3rd, and 4th years of compulsory secondary education (ESO, due to its initials in Spanish). Participants were required to be between 12 and 17 years old (*M* = 14.5, SD = 1.11) to meet the eligibility criteria. Initially, the sample consisted of 1,280 adolescents; however, 188 were excluded after data processing due to incomplete attendance, language barriers, or random response patterns (see Figure 2). The final sample comprised 1,092 adolescents, of whom 53.5% were female (*n* = 584; *M* = 14.14, SD = 1.04) and 46.5% were male (*n* = 508; *M* = 14.17, SD = 1.17). The distribution of the sample by academic year was as follows: 2nd ESO (*n* = 400; 194 males, 206 females), 3rd ESO (*n* = 342; 143 males, 199 females), and 4th ESO (*n* = 350; 171 males, 179 females).

### 2.2 Instruments

An *ad hoc* questionnaire was initially designed to collect sociodemographic data from the students, including age, sex, educational level, and country of origin. Additionally, other variables related to the school environment, academic performance, involvement in violent behaviors, and personal interactions with immediate social contexts—such as parents, legal guardians, teachers, and peers—were also assessed.

The HUMAS scale (Pérez-Fuentes et al., 2019a; Pérez-Fuentes et al., 2019b), which demonstrated a reliability of ⍺ = 0.75 in this study, is designed to assess humanization through 19 items rated on a five-point Likert scale (ranging from “never” to “always”). It comprises five subscales: (1) Willingness to optimism (α = 0.62) measured by items such as “I look forward to the future with enthusiasm”; (2) Sociability (α = 0.67) assessed through statements like “When I relate to my peers I try to put myself in their place”; (3) Emotional Understanding (α = 0.70) with items such as “When I dislike someone, I make an effort to understand them and give them a chance to get to know them”; (4) Self-Efficacy (α = 0.68) through statements like “I am satisfied with what I do and how I do it”; and (5) Affect (α = 0.73) evaluated using items such as “I feel nervous when I perform group tasks.”

The Maslach Burnout Inventory-Student Survey (MBI-SS) (Pérez-Fuentes et al., 2020) used in this study is the Spanish adaptation for adolescents based on the original Burnout scale by Maslach and Jackson (1981, 1986), demonstrating a reliability of α = 0.72. This instrument is designed to assess students’ feelings and attitudes toward their academic activities by measuring burnout in terms of its frequency and intensity. It consists of 12 items rated on a 7-point Likert scale (from 0, “Never” to 6, “Always/Every day”). The scale is divided into three subscales: (1) Emotional exhaustion (α = 0.83) through items such as “I feel emotionally drained by my studies”; (2) Depersonalization (α = 0.84) with items such as “I have become less enthusiastic about my studies”; and (3) Academic efficacy (α = 0.80) assessed with items like “In my opinion, I am a good student.”

The Family Functioning Scale (APGAR) (Bellón et al., 1996) used in this study is the Spanish adaptation of the original version by Smilkstein et al. (1982), demonstrating a reliability of α = 0.80. This scale consists of five items that evaluate key aspects of family functioning: adaptability, growth, partnership, affection, and resolve. Responses are measured on a three-point scale (0 = “Almost Never,” 1 = “Sometimes,” 2 = “Almost Always”) through items such as, “Are you satisfied with the time you and your family spend together?”

### 2.3 Procedure

Educational institutions in Almería offering Compulsory Secondary Education were randomly contacted. Initial communication with the responsible staff (administration or school office) was conducted via telephone or email, providing detailed information about the research project, its objectives, and the questionnaire booklet for student administration. Six high schools agreed to participate, and data collection took place during the first quarter of the 2023/24 academic year.

After obtaining permissions from the schools and informed consent from parents, visits were scheduled in coordination with the schools. Assessments were administered in classrooms with the presence of the respective academic tutor.

Each group session lasted 2 h; although the tests could be completed in approximately one-hour, two-hour weekly slots were scheduled for each group to allow for two sessions, minimizing fatigue and ensuring optimal performance. To clearly separate the sessions, a statement reading “End of Session 1” was included in the questionnaire, instructing students to stop at that point and conclude the first session. To maintain anonymity while ensuring participant recognition in the second session, students created an anonymous code to record in their notebooks. They were instructed to construct a code based on a pattern of personal cues that would be easy for them to remember.

Each session began with a thorough explanation of the study’s objectives, followed by clear instructions for participants to complete the questionnaires individually. Participants were assured of the privacy and secure statistical handling of their data, with an emphasis on the voluntary, anonymous, and confidential nature of their participation. No costs were incurred for participation, and no financial compensation was provided. The study was conducted in accordance with the World Medical Association’s Declaration of Helsinki, as it involved human subjects. Additionally, the research received approval from the Bioethics Committee of the University of Almería (Reference: UALBIO2020/046).

### 2.4 Data analysis

This cross-sectional study employed a quantitative, descriptive, and correlational design. Data analysis was conducted using IBM SPSS Statistics for Windows (Version 29) (IBM Corp, 2023). First, the reliability of the instruments used for data collection was assessed by estimating Cronbach’s alpha coefficient (Cronbach, 1951).

Pearson’s correlation coefficient was calculated to analyze the correlation among the study variables. The interpretation of the magnitude of the correlation followed Cohen’s (1988) guidelines: rxy < 0.3 indicating a weak correlation, 0.3 ≤ rxy < 0.5 indicating a moderate correlation, and 0.5 ≤ rxy indicating a strong correlation.

Additionally, a comparative analysis of means was performed to identify significant differences in personal skills, academic burnout, and family functioning based on sex, using Student’s t-test for independent samples. Cohen’s d was calculated to estimate effect sizes, following the author’s criteria: 0.2 (small effect), 0.5 (medium effect), and 0.8 (large effect) (Cohen, 1988).

Subsequently, simple mediation models were estimated, with the total score on personal competencies (HUMAS scale) as the independent variable, family functionality as the mediating variable, and each factor of academic burnout (emotional exhaustion, cynicism, and academic efficacy) as the dependent variable in separate models. The mediation models were computed using the PROCESS macro (v.4.0) for SPSS (Hayes, 2013), applying the bootstrapping technique with estimated coefficients from 5,000 bootstrap samples, and a 95% confidence interval.

## 3 Results

### 3.1 Humanization, burnout and family functionality: correlation analysis

As shown in Table 1, the correlation matrix of the humanization, burnout and family functioning variables is presented.

**Table 1:** Humanization, burnout and family functionality.

	Disposition to optimism	Sociability	Emotional understanding	Self-efficacy	Affectation	Emotional exhaustion	Cynicism	Academic efficacy	Family Functionality
Disposition to optimism		0.21**	0.11**	0.58**	0.29**	−0.27**	−0.25**	0.41**	0.43**
Sociability			0.44**	0.28**	−0.16**	−0.06*	−0.19**	0.19**	0.16**
Emotional understanding				0.20**	−0.17**	−0.12**	−0.15**	0.09**	0.06
Self-efficacy					0.22**	−0.26**	−0.30**	0.51**	0.38**
Affectation						−0.38**	−0.20**	0.20**	0.22**
Emotional exhaustion							0.57**	−0.20**	−0.23**
Cynicism								−0.28**	−0.27**
Academic efficacy									0.34**
Mean	10.63	10.99	7.95	18.35	13.44	14.06	9.75	14.62	7.75
SD	2.37	2.39	2.82	3.44	4.10	5.85	6.56	5.16	2.37
Min - Max.	3–15	3–15	3–15	5–25	5–25	0–24	0–24	0–24	0–10

Correlation matrix and descriptive statistics. *p < 0.05; **p < 0.01.

Positive correlations were found between family functionality and most dimensions of the HUMAS scale: Disposition to Optimism (*r* = 0.43; *p* < 0.01), Sociability (*r* = 0.16; *p* < 0.01), Self-Efficacy (*r* = 0.38; *p* < 0.01), and Affect (*r* = 0.22; *p* < 0.01). However, no correlation was observed with the Emotional Understanding dimension.

Similarly, Family Functionality was negatively correlated with two dimensions of Burnout: Emotional Exhaustion (*r* = − 0.23; *p* < 0.01) and Cynicism (*r* = − 0.27; *p* < 0.01), and positively correlated with Academic Efficacy (*r* = 0.34; *p* < 0.01).

Furthermore, all HUMAS dimensions were significantly correlated with each dimension of Academic Burnout, showing negative correlations with Emotional Exhaustion and Cynicism, and positive correlations with Academic Efficacy.

Next, a Student’s *t*-test for independent samples was performed to determine whether significant differences exist based on students’ sex.

### 3.2 Humanization, burnout and family functionality: mean comparison according to sex

Statistically significant sex differences were found in most dimensions of the HUMAS scale. Specifically, males reported higher mean scores than females in Disposition to Optimism (*t* = 6.94, *p* < 0.001, *d* = 0.42), Self-Efficacy (*t* = 6.62, *p* < 0.001, *d* = 0.40), and Affect (*t* = 9.57, *p* < 0.001, *d* = 0.58). Conversely, females scored higher than males in Sociability (*t* = − 4.35, *p* < 0.001, *d* = 0.26). No significant sex differences were observed in the Emotional Understanding dimension (*t* = −1.33, *p* = 0.091).

Regarding Burnout dimensions, significant differences were identified in Cynicism (*t* = 3.78, *p* < 0.001, *d* = 0.23), with females scoring higher, and in Academic Efficacy (*t* = 4.94, *p* < 0.001, d = 0.30), where males showed higher mean scores. No significant differences were found in Emotional Exhaustion (*t* = −1.13, *p* = 0.130), despite a higher mean in females.

Lastly, significant differences in Family Functionality were noted (*t* = 3.60, *p* < 0.001, *d* = 0.22), with males reporting a higher mean score than females (see Table 2).

**Table 2:** Humanization, burnout and family functionality.

		Sex						*t*	*p*	Confidence interval	Cohen’s *d*
		Man			Women						
		*N*	Mean	SD	*N*	Mean	SD				
HUMAS	Disposition to optimism	508	11.16	2.25	584	10.18	2.37	6.94	<0.001	0.70, 1.25	0.42
	Sociability	508	10.66	2.30	584	11.28	2.42	−4.35	<0.001	−0.91, −0.34	0.26
	Emotional understanding	508	7.83	2.77	584	8.06	2.87	−1.33	0.091	−0.56, 0.11	0.08
	Self-efficacy	508	19.07	3.15	584	17.72	3.56	6.62	<0.001	0.94, 1.75	0.40
	Affectation	508	14.67	3.95	584	12.38	3.93	9.57	<0.001	1.82, 2.76	0.58
MBI-SS	Emotional exhaustion	508	13.84	5.96	584	14.24	5.75	−1.13	0.130	−1.10, 0.30	0.07
	Cynicism	508	10.55	6.61	584	23.44	6.45	3.78	<0.001	0.72, 2.27	0.23
	Academic efficacy	508	15.44	4.91	584	13.91	5.27	4.94	<0.001	0.92, 2.14	0.30
	Family Functionality	508	8.02	2.16	584	7.51	2.52	3.60	<0.001	0.23, 0.79	0.22

Descriptives and *t*-test according to sex.

### 3.3 Mediation models

In the initial regression analysis, which is common across all three models, Family Functionality (M) was designated as the outcome variable, and the effect of the total score in Humanization competencies (X) was estimated, yielding a significant effect (*β* = 0.11, *p* < 0.001).

**Figure 3:**
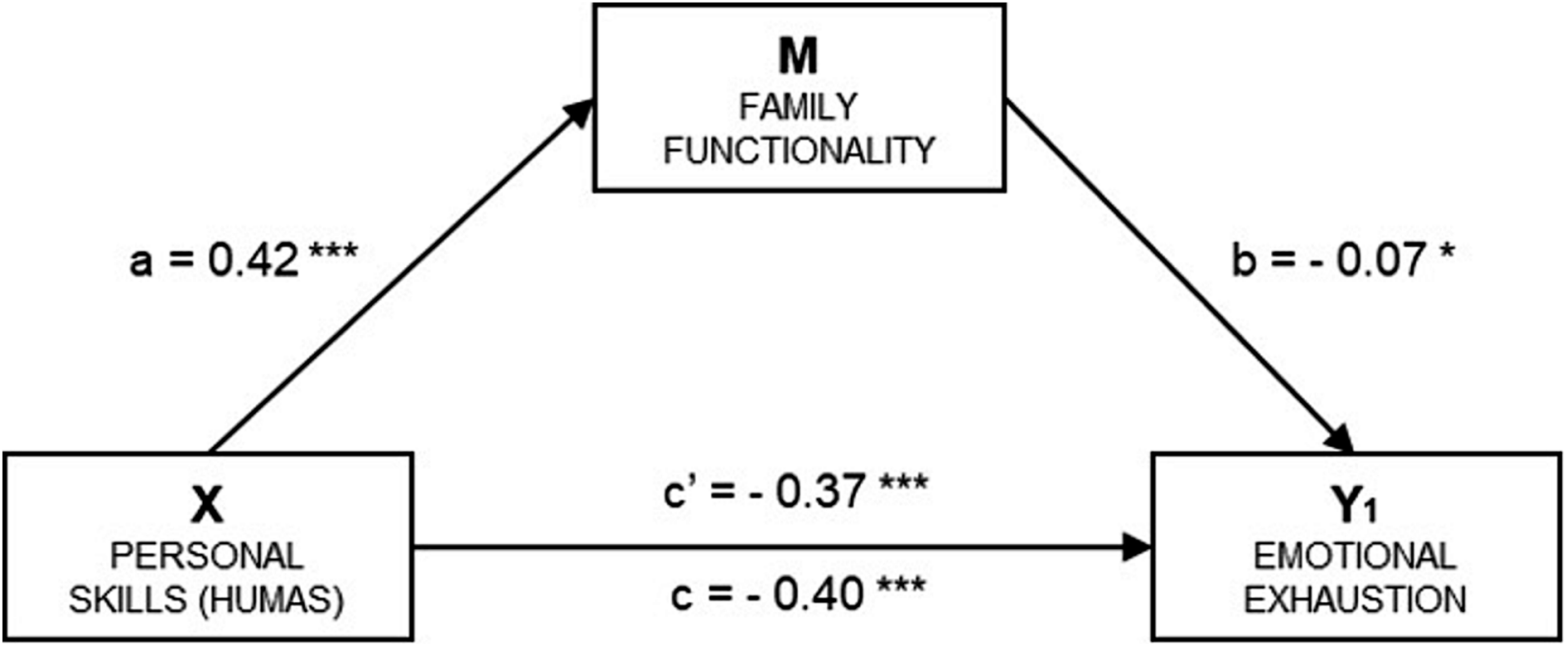
Simple mediation model of family functionality on the relationship between humanization and the emotional exhaustion of academic burnout. a = Direct effect of X on M; b = Direct effect of M on Y_1_; c' = Direct effect of X on Y_1_; c = Total effect of X on Y_1_. Indirect effect of X on Y_1_ through M: *β* = −0.03, SE = 0.013, 95% CI (−0.056, −0.006). Standardized coefficients are presented. Source: Own elaboration.

Table 3 and Figure 3 present the results of the simple mediation model for the Emotional Exhaustion factor of academic burnout (Y_1_). In the subsequent regression analysis, with Emotional Exhaustion (Y_1_) as the outcome variable, the direct effect of the independent variable was estimated (*β* = − 0.24, *p* < 0.001) along with the effect of the mediator (*β* = −0.18, *p* < 0.05). The total effect of the model was also significant (*β* = − 0.26, *p* < 0.001).

**Table 3:** Mediation estimates.

Effect	Label	Estimate	SE	95% CI		*p*	Standardized estimates
				Lower	Upper		
Indirect	a x b	−0.020	0.008	−0.036	−0.004	0.013	−0.031
Direct	c’	−0.239	0.020	−0.280	−0.198	< 0.001	−0.368
Total	c’ + a x b	−0.260	0.019	−0.297	−0.221	< 0.001	−0.399

Simple mediation model of family functionality on the relationship between humanization and the emotional exhaustion of academic burnout.

Finally, the analysis of indirect effects using the *bootstrapping* technique showed a significant result (*β* = −0.02, SE = 0.008, 95% CI - 0.037, −0.004), indicating a mediation effect of 7.78%. The standardized coefficients are displayed in the corresponding figure.

**Figure 4:**
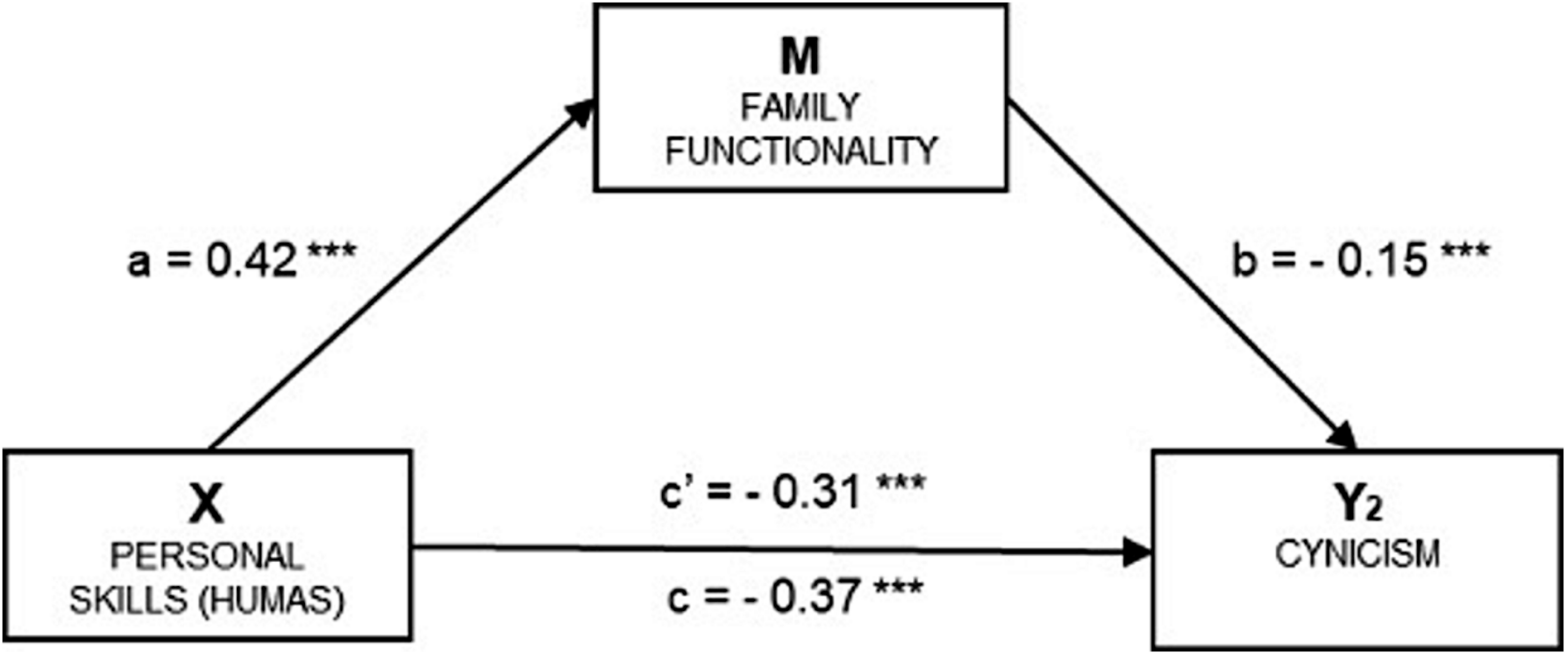
Simple mediation model of family functionality on the relationship between humanization and the cynicism of academic burnout. a = Direct effect of X on M; b = Direct effect of M on Y_2_; c' = Direct effect of X on Y_2_; c = Total effect of X on Y_2_. Indirect effect of X on Y_2_ through M: *β* = −0.06, SE = 0.014, 95% CI (−0.090, −0.034). Standardized coefficients are presented. Source: Own elaboration.

In Table 4 and Figure 4, Cynicism (Y_2_) is taken as the outcome variable in the regression model. The direct effect of the independent variable was estimated (*β* = −0.22, *p* < 0.001), along with the effect of the mediator (*β* = −0.41, *p* < 0.001), both of which were statistically significant. The total effect of the model was also significant (*β* = −0.27, *p* < 0.001). The analysis of indirect effects using the *bootstrapping* technique revealed a significant result (*β* = −0.04, SE = 0.010, 95% CI - 0.066, −0.025), indicating a mediation effect of 16.8%. The standardized coefficients are presented in the figure.

**Table 4:** Mediation estimates.

Effect	Label	Estimate	SE	95% CI		*p*	Standardized estimates
				Lower	Upper		
Indirect	a x b	−0.045	0.010	−0.066	−0.025	< 0.001	−0.062
Direct	c’	−0.223	0.023	−0.270	−0.178	< 0.001	−0.306
Total	c’ + a x b	−0.269	0.020	−0.309	−0.227	< 0.001	−0.368

Simple mediation model of family functionality on the relationship between humanization and the cynicism of academic burnout.

**Figure 5:**
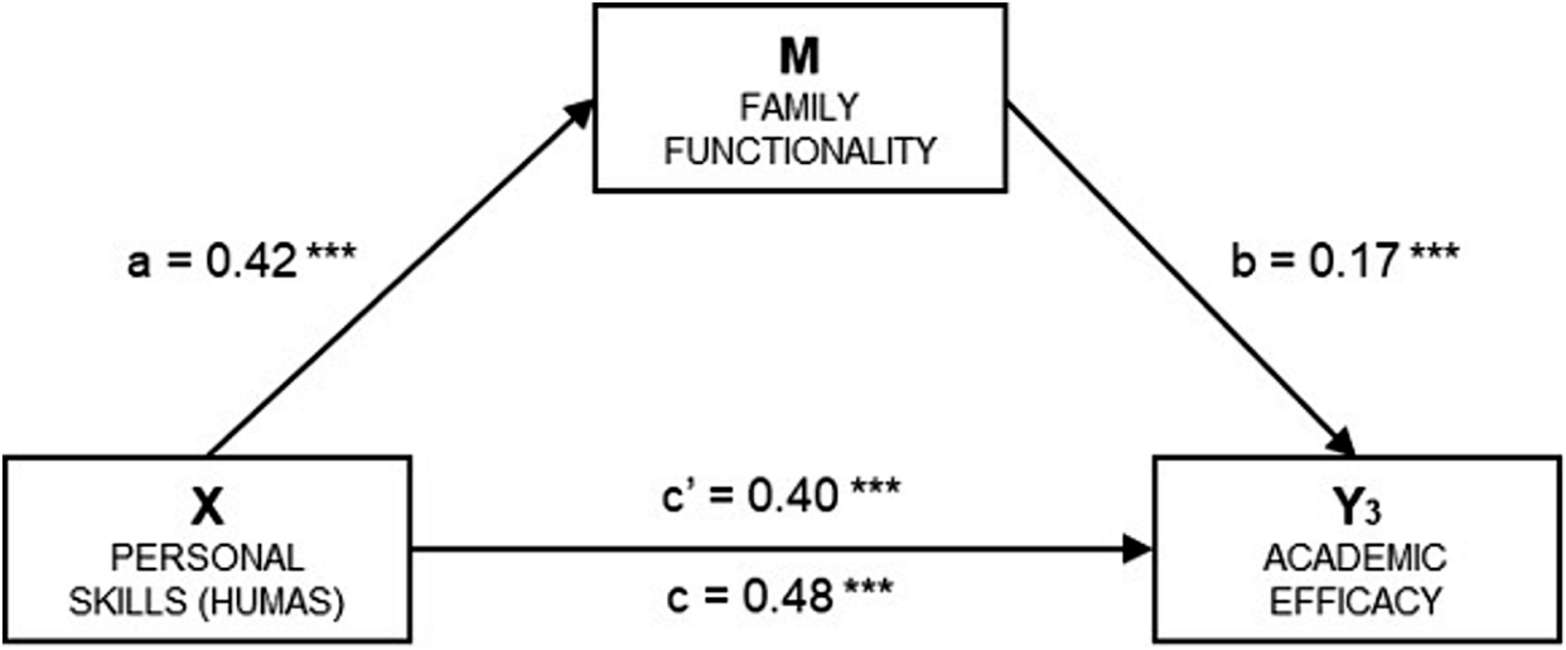
Simple mediation model of family functionality on the relationship between humanization and the academic efficacy of academic burnout. a = Direct effect of X on M; b = Direct effect of M on Y_3_; c' = Direct effect of X on Y_3_; c = Total effect of X on Y_3_. Indirect effect of X on Y_3_; through M: *β* = 0.07, SE = 0.014, 95% CI (0.047, 0.100). Standardized coefficients are presented. Source: Own elaboration.

Table 5 and Figure 5 illustrate the simple mediation model for the Academic Efficacy factor of Academic Burnout. In this model, Academic Efficacy (Y_3_) is designated as the outcome variable. The estimated effects of the independent variable (*β* = 0.23, *p* < 0.001) and the mediator (*β* = 0.38, *p* < 0.001) were both statistically significant, and the total effect of the model also reached significance (*β* = 0.27, *p* < 0.001). The analysis of indirect effects yielded a significant result (*β* = 0.04, SE = 0.008, 95% CI 0.027, 0.058), indicating a mediation effect of 15.3%. The standardized coefficients are presented in the corresponding figure.

**Table 5:** Mediation estimates.

Effect	Label	Estimate	SE	95% CI		*p*	Standardized estimates
				Lower	Upper		
Indirect	a x b	0.042	0.007	0.027	0.058	< 0.001	0.073
Direct	c’	0.231	0.017	0.197	0.265	< 0.001	0.403
Total	c’ + a x b	0.273	0.016	0.241	0.305	< 0.001	0.476

Simple mediation model of family functionality on the relationship between humanization and the academic efficacy of academic burnout.

## 4 Discussion

The aim of this study was to analyze the relationships between humanization competencies, academic burnout, and family functioning in secondary school students aged 12 to 16. In addition, the study sought to examine potential sex differences in these variables, including humanization competencies, academic burnout, and family functioning. Finally, the research aimed to investigate the mediating role of family functioning in the relationship between humanization competencies and academic burnout among the adolescent participants.

Among personal humanization competencies, Disposition to Optimism, emotional skills, Sociability, Affectivity, and Self-Efficacy stand out, as pointed out by Pérez-Fuentes et al. (2019a, 2019b). These competencies should be supported by appropriate family and educational functionality during adolescence to strengthen and optimize individual development (Márquez and Gaeta, 2017). Inadequate development of these competencies has been associated with poorer academic performance and heightened classroom stress in adolescent students (Llanos, 2016; Pérez and Chávez, 2024).

Persistent stress in the school environment can contribute to the onset of academic burnout syndrome, which is defined as a negative psychological state experienced by students in relation to their learning processes (Gao, 2023). The development of this syndrome is influenced by both individual and family factors (Liu et al., 2024; Zhang et al., 2023).

Based on the above, the first hypothesis was established, with results supporting the existence of a positive correlation between adolescent family functionality and nearly all personal humanization competencies, except for Emotional Understanding. Similarly, family functionality was found to positively correlate with academic burnout dimensions, such as Academic Efficacy, while showing a negative correlation with Emotional Exhaustion and Cynicism. Moreover, significant correlations were observed between all dimensions of the HUMAS scale and those of academic burnout, indicating that all the dimensions of the former are negatively associated with Emotional Exhaustion and Cynicism, and positively associated with Academic Efficacy.

Statistically significant sex differences were observed across most dimensions of the three scales. Regarding personal humanization competencies, males showed higher mean scores than females in Disposition to Optimism, Self-Efficacy, and Affect. Conversely, females scored higher in Sociability. No significant differences were found in the Emotional Understanding dimension. These findings align with previous research, which reported no sex differences in the mean scores of individual dimensions or the overall scale (Bermejo et al., 2013; Villacieros et al., 2019).

The comparison by sex for the burnout variable in this study revealed significant differences in Cynicism, with females showing a higher mean score, whereas males scored higher in Academic Efficacy. No significant differences were found in Emotional Exhaustion, although the mean was higher among female adolescents. These findings are consistent with previous research, which suggests that academic burnout is prevalent in both sexes but tends to yield higher scores in girls (Read et al., 2022; Vinter et al., 2021).

The observed gender differences in humanization and burnout dimensions have important implications for the development of targeted interventions. Higher scores in sociability among women and in optimism, self-efficacy, and affectivity among men may reflect gendered socialization patterns that influence how both genders manage emotional and academic competencies. Additionally, the higher cynicism scores in women and greater academic efficacy in men suggest that burnout experiences may be shaped by societal expectations and gender roles, which could impact the design of support and prevention strategies in these areas.

Finally, the results indicated significant sex differences in family functionality, with males showing a higher mean score than females. This finding is consistent with the study by Barragán et al. (2021), which also reported higher family functionality scores in males. In addition to these results, Spitz and Steinhausen (2023) reported lower parental cohesion scores in male adolescents compared to females, supporting the second hypothesis of this study.

Regarding the third hypothesis, the results confirm that family functionality serves as a mediator in the relationship between personal competencies (HUMAS) and the dimensions of academic burnout in adolescents. Specifically, family functionality exhibited the strongest mediating effect with Cynicism, followed by Academic Efficacy and Emotional Exhaustion. This suggests that higher levels of humanization competencies in adolescents are associated with lower levels of emotional exhaustion and cynicism, and greater academic efficacy, through the positive influence of family functionality. These findings align with previous research, which also identified family functionality as a key mediator when examining similar variables (González and Molero, 2023).

The results suggest that schools should integrate programs to develop humanization competencies into their curricula. Activities fostering socio-emotional learning could reduce academic burnout. For families, promoting functional dynamics—such as open communication and emotional support—can help mitigate stress and improve adolescents’ academic well-being. Parental training programs focused on cohesion and adaptability may be particularly effective. Strengthening collaboration between schools and families is also essential to maximize these benefits.

## 5 Conclusion

The significant results of this study aim to elucidate the mediating role of family functionality, based on adolescents’ humanization competencies, and its impact on academic burnout, thereby providing a deeper understanding of these interrelated factors. Despite the insights gained, several limitations were identified. One major limitation concerns the type of assessments used, as self-report instruments are susceptible to social desirability bias, wherein adolescents may unconsciously respond in a manner that reflects a more favorable self-image. Although instructions were provided to mitigate this effect, such limitations are inherent to this type of data collection.

Another limitation relates to timing constraints, as some sessions were conducted when participants had low motivation or competing priorities, such as impending exams. Additionally, although the assessments were intended to be completed individually, some participants occasionally shared their responses with peers seated nearby. Language barriers were also a challenge for some adolescents, which hindered their understanding of certain items and required additional time and effort to complete the questionnaires. Furthermore, the contextualization of the present study was constrained by a lack of prior research examining the three variables - humanization competencies, academic burnout, and family functionality - simultaneously in adolescents. Although this study establishes the mediating role of family functionality between humanization competencies and academic burnout, the underlying mechanisms require further exploration. A functional family environment could enhance adolescents’ emotional regulation and coping strategies, mitigating academic stress and promoting academic efficacy. Future studies could use longitudinal designs to clarify whether improvements in family functionality led to sustained development of humanization competencies and a reduction in burnout. Additionally, qualitative methods such as interviews could provide deeper insights into how family dynamics influence adolescents’ academic and emotional well-being. Related studies exist, but they have typically explored these variables in isolation, supporting the relevance and contribution of this research. Also, the sample’s limitation to adolescents from a single spanish province may affect the generalizability of the findings, as cultural and geographical differences could influence how academic burnout, and related factors manifest in other regions.

This study addresses a gap in the literature by highlighting the mediating role of family functioning in the relationship between personal competencies and academic burnout, offering new insights into adolescent well-being. These findings underscore the need for practical applications, such as family-focused intervention programs that enhance communication and cohesion, alongside school-based initiatives to strengthen students’ social–emotional skills. This can serve as a foundation for educational professionals and parents to better understand how adolescents’ family functioning and personal competencies influence their academic well-being and mental health. It is recommended that these results be used to inform the design of intervention programs aimed at fostering a healthy family environment and enhancing social–emotional skill development in adolescents, which would, in turn, help prevent academic burnout and promote improved academic performance and overall well-being.

In conclusion, efforts should focus on promoting comprehensive educational approaches that incorporate these intervention programs into the academic curricula of schools, both in Spain and globally, adapting their objectives to the specific needs and characteristics of the students and educational staff involved.

## Data Availability

The data presented in this study are available on request from the corresponding author.
